# Supplementary material for: Huperzine—A Improved Animal Behavior in Cuprizone-Induced Mouse Model by Alleviating Demyelination and Neuroinflammation
Source: Int J Mol Sci. 2022 Dec 19;23(24):16182. doi: 10.3390/ijms232416182 (PMC9785798; doi:10.3390/ijms232416182)
Supplement: Supplementary file 1 [file ijms-23-16182-s001.zip › ijms-1949960-Supplementary_Material.pdf]

Supplementary Material

1. HupA Promoted the Formation of New Myelin Sheath in Demyelinated Mice

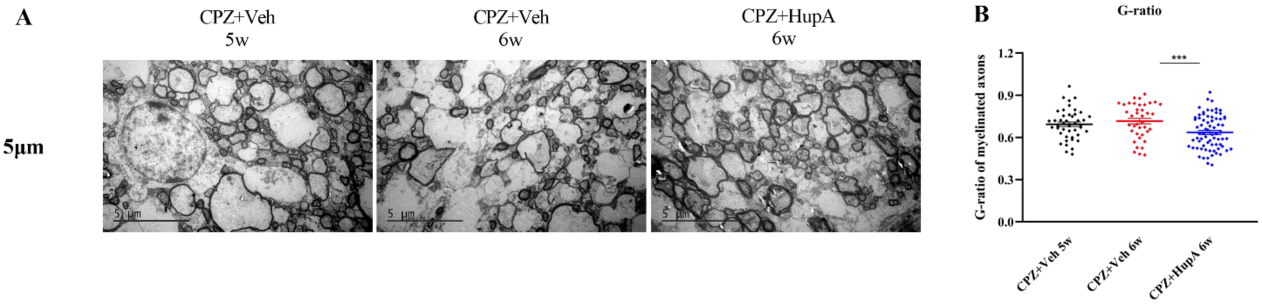

Supplementary Figure S1. HupA treatment regulated the remyelination in the corpus callosum. (**A**) TEM ultrastructure of the corpus callosum at week 5 and week 6 (Scale bar=5 μm). (**B**) Each group of g-ratio in the corpus callosum. The results represent the mean ± SEM (***p < 0.001, n = 4 mice per group).

2.HupA Regulated the Activation of Microglia in Hippocampus of Demyelinated Mice

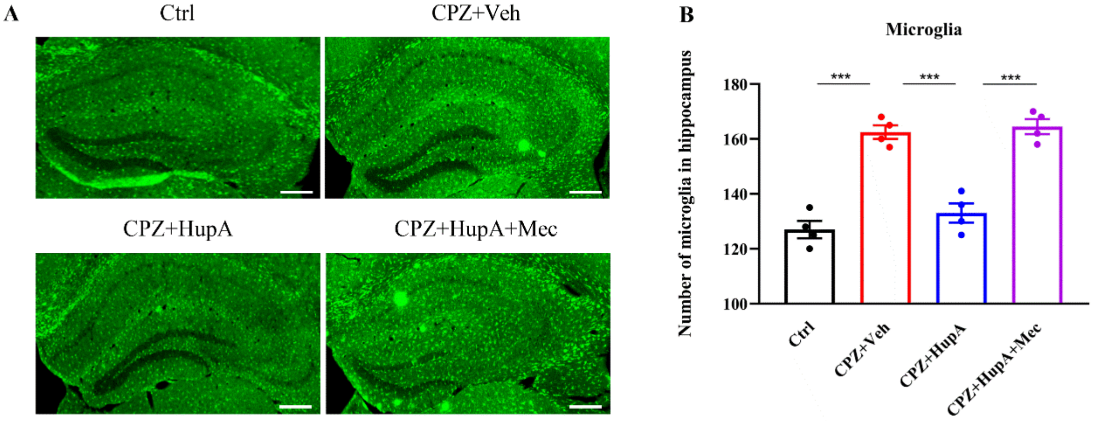

Supplementary Figure S2. HupA treatment reduced the quantity of microglia in hippocampus. (**A**) Iba1 immunofluorescence staining. (**B**) Quantitative analysis of microglia in hippocampus. The results represent the mean ± SEM (***p < 0.001, n = 4 mice per group).
